# In Situ Peroxidase Labeling Followed by Mass-Spectrometry Reveals TIA1 Interactome

**DOI:** 10.3390/biology11020287

**Published:** 2022-02-11

**Authors:** Olga Gourdomichali, Katerina Zonke, Fedon-Giasin Kattan, Manousos Makridakis, Georgia Kontostathi, Antonia Vlahou, Epaminondas Doxakis

**Affiliations:** 1 Center of Basic Research, Biomedical Research Foundation, Academy of Athens, 11527 Athens, Greece; ogourdom@bioacademy.gr (O.G.); katerinazonke@gmail.com (K.Z.); fkattan@bioacademy.gr (F.-G.K.); mmakrid@bioacademy.gr (M.M.); gkontostathi@bioacademy.gr (G.K.); vlahoua@bioacademy.gr (A.V.); 2 Department of Biology, National and Kapodistrian University of Athens (NKUA), 15784 Athens, Greece; 3 Department of Biological Applications and Technology, Faculty of Health Sciences, University of Ioannina, 45110 Ioannina, Greece

**Keywords:** TIA1, APEX2, proximity labeling, proteomics, LC-MS/MS, RNA binding proteins, stress, stress granules

## Abstract

**Simple Summary:**

T-cell intracellular antigen 1 (TIA1) is a DNA/RNA-binding protein best known for its different roles in RNA metabolism. Currently, little is known about the interacting protein partners of TIA1 in control and stress conditions that could shed light on its multiple context-specific molecular functions. Proximity labeling is a technique in which a labeling enzyme, here APEX2, that is fused to the protein of interest, in this case TIA1, marks the protein’s interacting network in living cells, allowing for subsequent ex vivo analysis using protein identification methods such as mass spectrometry. Hereby, combining these two techniques, it was revealed that the TIA1 interactome has very distinct protein partners in control and unstressed cells and that these partners are involved in not only previously identified processes such as splicing, nucleocytoplasmic transport, and different levels of protein translation control, but also in novel ones such as DNA double-strand break repair and mitochondrial metabolism. Overall, these findings provide a more precise definition of TIA1’s function in cells and pave the way to dissect its role in each of these processes.

**Abstract:**

TIA1 is a broadly expressed DNA/RNA binding protein that regulates multiple aspects of RNA metabolism. It is best known for its role in stress granule assembly during the cellular stress response. Three RNA recognition motifs mediate TIA1 functions along with a prion-like domain that supports multivalent protein-protein interactions that are yet poorly characterized. Here, by fusing the enhanced ascorbate peroxidase 2 (APEX2) biotin-labeling enzyme to TIA1 combined with mass spectrometry, the proteins in the immediate vicinity of TIA1 were defined in situ. Eighty-six and 203 protein partners, mostly associated with ribonucleoprotein complexes, were identified in unstressed control and acute stress conditions, respectively. Remarkably, the repertoire of TIA1 protein partners was highly dissimilar between the two cellular states. Under unstressed control conditions, the biological processes associated with the TIA1 interactome were enriched for cytosolic ontologies related to mRNA metabolism, such as translation initiation, nucleocytoplasmic transport, and RNA catabolism, while the protein identities were primarily represented by RNA binding proteins, ribosomal subunits, and eicosanoid regulators. Under acute stress, TIA1-labeled partners displayed a broader subcellular distribution that included the chromosomes and mitochondria. The enriched biological processes included splicing, translation, and protein synthesis regulation, while the molecular function of the proteins was enriched for RNA binding activity, ribosomal subunits, DNA double-strand break repair, and amide metabolism. Altogether, these data highlight the TIA1 spatial environment with its different partners in diverse cellular states and pave the way to dissect TIA1 role in these processes.

## 1. Introduction

T-cell intracellular antigen 1 (TIA1) is a prion-like RNA-binding protein (RBP), best characterized for its pleiotropic roles in RNA metabolism. In control conditions, it resides in both the nucleus and cytosol. In the nucleus, TIA1 regulates transcription [1,2] and pre-mRNA splicing [3,4,5,6,7]. In the cytoplasm, similar to classical RBPs, TIA1 binds to U-rich motifs of mature mRNAs to regulate their localization, translation, and stability [8,9,10]. In response to cellular stress, TIA1 localizes almost exclusively to the cytoplasm to suppress mRNA translation by binding to its mRNA targets and, importantly, nucleating stress granule (SG) formation [9,11,12]. Interestingly, while other RBPs (e.g., IGF2BP1 or HUR), which are dispensable for SG-assembly, are stably associated with SGs, TIA1 is only transiently associated with SGs, promoting SG-formation by constantly replenishing mRNPs [13]. Missense mutations in the *TIA1* gene, responsible for Welander distal myopathy (WDM) [14] and amyotrophic lateral sclerosis (ALS), are characterized by delayed SG disassembly and accumulation of non-dynamic SGs that harbor cytotoxic TAR DNA-binding protein 43 (TARDBP, TDP43) [15].

The importance of TIA1 in animal physiology has been revealed by multiple in vivo and in vitro studies. Half of the mice deficient for TIA1 die perinatally, while those that remain alive develop inflammation in different tissues [16,17]. In the murine brain, TIA1 regulates stress-dependent synaptic plasticity and fear memory by regulating the expression of immune system genes with modulatory roles in the hippocampus [18]. Transcriptome profiling of TIA1 knockout brains further revealed alterations in the expression of cell cycle and apoptotic markers and the deregulation of fat storage and membrane trafficking factors [19]. Cell culture studies have provided additional evidence of the role of TIA1 in cell homeostasis. In MEF cells, TIA1 deficiency leads to metabolic dysregulation, reduced cell proliferation rates, cell cycle progression delay, increased cell size, and a moderate increase in cell death [20]. In HEK293 cells, TIA1 overexpression partially represses global translation and, similar to MEF knockouts, drives cell-cycle arrest and caspase-dependent apoptosis [21]. Moreover, TIA1 overexpression affects mitochondria by enhancing fission, ROS production, and mitochondrial DNA damage by stabilizing and alternatively splicing MFF mRNA and OPA pre-mRNA, respectively [22,23].

TIA1 harbors a C-terminal prion-related domain, rich in the polar amino acids asparagine and glutamine, necessary to drive protein aggregation and SG assembly. Yet, unlike conventional prions and prion-related proteins associated with severe forms of neurodegeneration phenotype, TIA1 binding to other proteins serves physiological processes positively [24]. Currently, we know little about TIA1′s interacting partners in control and stress conditions, which limits our understanding of its molecular functions. Proximity labeling is a technique in which a labeling enzyme fused to the protein of interest (also known as ‘bait’) marks the protein’s interaction network in vivo, enabling subsequent ex vivo analysis. The overarching advantage of this technique is that the labeling is performed in living cells when all of its compartments are intact, and unlike biochemical methods such as co-immunoprecipitation (co-IP) and protein pull-down that rely on direct and stable interactions, the technique can also capture transient and dynamic interactions. Here, we linked the highly-active, second-generation ascorbate peroxidase (APEX2) onto TIA1 to perform proximity labeling [25]. APEX2 catalyzes, in the presence of hydrogen peroxide, the conversion of a cell-permeable, biotin-tyramide substrate into short-lived (<1 ms) highly-reactive biotin-phenoxyl radicals that label aromatic amino acids in proteins within ~20 nm of the enzyme. Using this technique, we labeled all TIA1 partners in control and acute stress conditions. Subsequently, we carried out mass spectrometry (MS) and validation (co-IP and immunoblotting) studies to identify them.

## 2. Materials and Methods

### 2.1. Antibodies

The rabbit polyclonal anti-PCMT1 (10519-1-AP), anti-RPL7L1 (16707-1-AP), anti-TOMM40 (18409-1-AP), anti-MRPL15 (18339-1-AP), anti-FMRP (13755-1-AP), anti-HADHB (29091-1-AP), anti-PMPCB (16064-1-AP), anti-MSH2 (15520-1-AP), anti-FEN1 (14768-1-AP), anti-RPA2 (10412-1-AP), anti-PHB2 (12295-1-AP), anti-IGF2BP1 (22803-1-AP), anti-MCCC1 (14861-1-AP), anti-SMC3 (14185-1-AP), anti-HSD17B4 (15116-1-AP), anti-PRDX3 (10664-1-AP), anti-PCCA (21988-1-AP), anti-MCM2 (10513-1-AP), anti-MCM4 (13043-1-AP), anti-CUL4B (12916-1-AP), and anti-FUBP1 (24864-1-AP) were from Proteintech (Chicago, IL, USA); the mouse monoclonal anti-GAPDH (sc-365062), rabbit polyclonal anti-TARDBP (sc-102127) and goat polyclonal anti-TIA1 (sc-1751) antibodies were obtained from Santa Cruz Biotechnology, (Santa Cruz, Dallas, TX, USA); the horse anti-goat FITC-conjugated secondary antibody (DyLight 488, #DI-3088) was from Vector Labs (Burlingame, CA, USA)*;* the streptavidin-Alexa conjugate (#S11226) was from ThermoFisher (Waltham, MA, USA); the mouse (#7076) and rabbit (#7074) HRP-conjugated secondary antibodies were from Cell Signaling Technologies (Danvers, MA, USA).

### 2.2. Generation of DNA Constructs

We amplified the human TIA1 CDS and APEX2 CDS (without FLAG and NES sequences) by PCR, using the proofreading Phusion polymerase (ThermoFisher) (for primer sequences, see Supplementary Table S1) from human SK-N-SH cells and pcDNA3 FLAG-APEX2-NES plasmid (Addgene # 49386), respectively. The PCR products were cloned using the HiFi system (NEB, Ipswich, MA, USA) between the HindIII and BamHI restriction sites of the paavCAG-pre-mGRASP plasmid (Addgene # 34911). We prepared three TIA1 fusion proteins that contained the full-length sequences of TIA1 and APEX2 with either short flexible (flexible 1: (GGGS)3 and flexible 2: GSAGSAAGSGEF) or rigid (GGAEAAAKEAAAKAAPAEAAAKEAAAKA) linker sequences in between. Sanger-sequencing verified the DNA sequence of all constructs (CeMIA SA, Larisa, Greece).

### 2.3. In Situ Labeling of TIA1 Interactors Mediated by APEX2-Mediated Biotinylation

Neuroblastoma SK-N-SH and human embryonic kidney (HEK) 293 cells were grown in high-glucose DMEM (#D6429, Sigma-Aldrich, St. Louis, MO, USA) supplemented with 10% fetal bovine serum (FBS) (#16000044, ThermoFisher) and 1% penicillin/streptomycin (#P4333, Sigma-Aldrich). Cells were kept at 37 °C in a humidified 5% CO_2_ incubator (ThermoFisher). SK-N-SH cells were transfected at the time of plating with APEX2 alone or TIA1-APEX2 hybrid expression plasmids by using the JetOptimus reagent (Polyplus, Illkirch, France). Thirty-six hours after transfection, cells were incubated in 1 mM biotin tyramide (#LS-3500, Iris Biotech, Marktredwitz, Germany) and 330 μM NaAsO_2_ (#S7400, Sigma-Aldrich) (reduced to 100 μΜ when used for immunofluorescence staining due to the cells’ poor adhesion at higher concentrations), where appropriate, for 45 min at 37 °C. After that, H_2_O_2_ was added at a final concentration of 1 mM for exactly 1.5 min at room temperature (RT). The reaction was quenched by adding Trolox (sc-200810) and sodium ascorbate (sc-215877) (both from Santa Cruz Biotechnology) dissolved in PBS to a final concentration of 5 mM and 10 mM, respectively. Cells were washed twice more with quenching solution and either fixed for 15 min with 4% (*w*/*v*) paraformaldehyde (#A11313, Alfa Aesar, Haverhill, MA, USA) for immunofluorescent staining or lysed with RIPA solution (see below) for the affinity capture assay.

### 2.4. Immunofluorescence Staining

PBS containing 0.5% Triton X-100 was used to permeabilize fixed SK-N-SH cells grown on poly-D-lysine-treated coverslips for 10 min at RT. Cells were then incubated for one hour at RT in a blocking solution containing 3% BSA and 0.02% Triton X-100 in PBS (PBST) and then probed overnight at 4 °C with a primary antibody against TIA1 diluted in blocking solution at 1:50 in a hybridization chamber. The following day, the cells were washed three times in PBS and incubated for one hour at RT with either a horse anti-goat FITC-conjugated secondary antibody (diluted at 1:500) or a Streptavidin-Alexa conjugate (diluted at 1:2000) in blocking solution (1% BSA in PBS). After two rounds of PBS washing, the cells were stained with DAPI (#D9542, Sigma-Aldrich) for 3 min, followed by two further rounds of PBS washing. Coverslips were mounted on slides using Vectashield (#H-1700, Vector Labs). Confocal imaging was performed using a Leica inverted confocal laser scanning microscope. Images were acquired using Leica LAS AF software through a 60× oil-immersion objective lens. All fluorescence photos from the various sample groups were taken using the same settings.

### 2.5. Preparation of Whole Protein Extracts and Affinity Capture of Biotinylated Proteins

Following the APEX2 labeling reaction, SK-N-SH cells were washed twice with quenching solution and resuspended in 200 μL ice-cold RIPA lysis buffer comprised of 25 mM Tris pH 7.5, 150 mM NaCl, 1.5 mM EDTA, 1% Triton X-100, 0.16% Sodium Deoxycholate, 0.16% SDS, and supplemented with 1.5 mM EDTA, 5 mM Trolox, 10 mM L-ascorbate and protease inhibitors (cOmplete, Roche, Basel, Switzerland). After incubation for 30 min in a rotor at 4 °C, cell suspensions were centrifuged for 15 min at 14,000 rcf at 4 °C, and the supernatants (whole-cell lysates) were transferred into new tubes. The Bradford Assay was used to determine protein concentration (BioRad Laboratories, Richmond, CA, USA).

Two washes of RIPA lysis buffer were used to equilibrate streptavidin magnetic particles (#11641786001, Roche). Each lysate was incubated for 2 h at RT with 90 μL of bead slurry in microcentrifuge tubes with rotation. Following that, the beads were washed twice with 1 mL RIPA lysis buffer, once with 1 mL 1 M KCl, once with 1 mL 100 mM sodium carbonate, twice with 1 mL 2 M Urea, and twice with 1 mL RIPA lysis buffer. Biotinylated proteins were then eluted by incubating the bead slurry for 15 min at RT with 100 μL of 2 M Thiourea, 6 M Urea, 1% SDS, 3 mM Biotin in ddH_2_O, followed by another 15 min at 98 °C.

### 2.6. Western Blot Analysis

Immunoblotting was carried out as previously described [26]. Briefly, equal amounts of whole-cell extracts or equal volumes of pull-down material were separated by 12% SDS-PAGE under denaturing conditions and transferred to nitrocellulose membrane (Protran; Amersham/Merck, St. Louis, MO, USA). The nitrocellulose membranes were probed with the appropriate primary antibodies after blocking with Tris-buffered saline (TBS) containing 5% nonfat milk and 0.1% Tween-20 for 1 h at RT. Except for the neutravidin-HRP conjugate diluted to a final concentration of 1:2000, all primary antibodies were diluted in blocking buffer at 1:1000. Secondary HRP-conjugated antibodies were used in a 1:2000 dilution. The immunoreactive bands were visualized with the enhanced chemiluminescence (ECL) method using the Clarity substrate (BioRad). Data obtained from at least three independent experiments are presented here.

### 2.7. Proteomics Analysis

Sample preparation: biotinylated proteins were eluted after an APEX labeling reaction with a buffer composed of 2 M Thiourea, 6 M Urea, 1% SDS, 3 mM Biotin, as mentioned above. Samples were subjected to buffer exchange (with 50 mM NH_4_HCO_3_) and at the same time concentrated to a final volume of 20 μL by utilizing Amicon centrifugation filters with 3 kDa molecular weight cutoff (MWCO). All the available volume of each sample (20 μL) was analyzed in SDS-PAGE (5% stacking, 12% separating) with the GeLC-MS method as previously described [27]. Briefly, electrophoresis was stopped when samples just entered the separating gel. Gels were fixed with 30% methanol, 10% acetic acid for 30 min, followed by 3 washes with water (3 × 10 min) and stained with Coomassie colloidal blue overnight. Excess of stain was washed with water (3 × 10 min washes). Each band was excised from the gel and further sliced into small pieces (1–2 mm). Gel pieces were destained with 40% Acetonitrile, 50 mM NH_4_HCO_3,_ and then reduced with 10 mM DTE in 100 mM NH_4_HCO_3_ for 20 min at RT. After reduction, samples were alkylated with 54 mM Iodoacetamide in 100 mM NH_4_HCO_3_ for 20 min at RT in the dark. Samples were then washed with 100 mM NH_4_HCO_3_ for 20 min at RT, followed by another wash with 40% Acetonitrile, 50 mM NH_4_HCO_3_ for 20 min at RT, and a final wash with ultrapure water under the same conditions was performed. Gel pieces were dried in a centrifugal vacuum concentrator (speed vac) and trypsinized overnight in the dark at RT by adding 600 ng of trypsin per sample (trypsin stock solution: 10 ng/μL in 10 mM NH_4_HCO_3_, pH 8.5). Peptides were extracted after incubation with the following buffers: 50 mM NH_4_HCO_3_ for 15 min at RT followed by two incubations with 10% Formic Acid, Acetonitrile (1:1) for 15 min at RT. Peptides were eluted in a final volume of 600 μL and filtered with 0.22 μm PVDF filters (Merck Millipore) before being dried in a centrifugal vacuum concentrator (speed vac). Dried peptides were reconstituted in mobile phase A buffer (0.1% formic acid, pH 3) and processed with LC-MS/MS analysis [27].

LC-MS/MS analysis: samples were resuspended in 10 μL mobile phase A (0.1% FA). A 5 μL volume was injected into a Dionex Ultimate 3000 RSLS nanoflow system (Dionex, Camberly, UK) configured with a Dionex 0.1 × 20 mm, 5 μm, 100 Å C18 nano trap column with a flow rate of 5 µL/min. The analytical column was an Acclaim PepMap C18 nano column 75 μm × 50 cm, 2 μm, 100 Å with a flow rate of 300 nL / min. The trap and analytical column were maintained at 35 °C. Mobile phase B was 100% ACN: 0.1% Formic acid. The column was washed and re-equilibrated prior to each sample injection. The eluent was ionized using a Proxeon nanospray ESI source operating in positive ion mode. A Q Exactive Orbitrap (Thermo Finnigan, Bremen, Germany) was operated in MS/MS mode for mass spectrometry analysis. The peptides were eluted under a 120 min gradient from 2% (B) to 33% (B). Gaseous phase transition of the separated peptides was achieved with positive-ion electrospray ionization applying a voltage of 2.5 kV. For every MS survey scan, the top 10 most-abundant multiply-charged precursor ions between m/z ratio 300 and 2200 and intensity threshold 500 counts were selected with FT mass resolution of 70,000 and subjected to HCD fragmentation. Tandem mass spectra were acquired with an FT resolution of 35,000. The normalized collision energy was set to 33, and already targeted precursors were dynamically excluded for further isolation and activation for 15 s with 5 ppm mass tolerance.

MS data processing: raw files were analyzed with Proteome Discoverer 1.4 software package (Thermo Finnigan), using the Sequest search engine and the Uniprot human (Homo sapiens) reviewed database, downloaded on 15 December 2017, including 20,243 entries. The search was performed using carbamidomethylation of cysteine as static and oxidation of methionine as dynamic modifications. Two missed cleavage sites, a precursor mass tolerance of 10 ppm and fragment mass tolerance of 0.05 Da were allowed. False discovery rate (FDR) validation was based on q value: target FDR of 0.01.

### 2.8. Co-Immunoprecipitation

Forty-eight hours following transfection of 1.5 × 10^7^ SK-N-SH cells with TIA1 expressing plasmid, cells were resuspended in 2 mL of ice-cold non-denaturing PLB lysis buffer containing 10 mM HEPES pH 7.0, 100 mM KCl, 5 mM MgCl_2_, 0.5% NP-40, and protease inhibitors. After incubation for 30 min in a rotor at 4 °C, the cell suspension was centrifuged for 15 min at 13,000 rpm at 4 °C, and the supernatant was transferred into new tubes. Half of the supernatant was then incubated overnight with 2 μg of either anti-goat IgG or anti-TIA1 primary antibodies at 2–8 °C with continuous mixing.

The Magnetic PureProteome™ A/G Mix bead suspension (Merck/Millipore, Burlington, MA, USA) was washed three times in PBS with 0.1% Tween-20 before being blocked for an hour at RT in PBS with 2% BSA and 0.1% Tween-20. After three PBS washes, the beads were equilibrated using NT2 washing/elution buffer composed of 50 mM Tris-HCl pH 7.4, 250 mM NaCl, 1 mM MgCl_2_, and 0.05% Tween-20. Each lysate was incubated in microcentrifuge tubes with 30 μL of bead slurry for 30 min at RT with rotation. After that, the beads were washed three times with NT2 buffer. The immunoprecipitated proteins were then eluted by incubating the bead slurry for 10 min at 98 °C in 100 μL of NT2 buffer supplemented with 6× Laemmli buffer comprised of 6% SDS, 30% β-mercaptethanol, 40% glycerol, and 0.005% bromophenol blue.

### 2.9. Gene Ontology Analysis

WebGestalt (WEB-based GEne SeT AnaLysis Toolkit) [28] was used to analyze Gene Ontology (GO) cellular component, molecular function, and biological process analyses with Benjamini-Hochberg multiple test correction (FDR of 0.05). The reference was the Homo sapiens genome protein-coding database.

The Cytoscape plugin ClueGo [29] was used with an FDR of 0.05 to show non-redundant biological words in a functional grouping network. The ClueGO network is created with kappa statistics and reflects the relationships between the terms based on the similarity of their associated genes. The node color is switched between functional groups and clusters distribution on the network. Related terms which share similar associated genes were fused to reduce redundancy.

### 2.10. Statistical Analysis

Data are presented as mean ± standard error of the mean (SEM) from at least three independent experiments (biological replicates). Comparisons were carried out using a *t*-test. The threshold for statistically significant differences was set to *p* < 0.05. Statistical analysis was performed using GraphPad Prism (Release 8.0.1, San Diego, CA, USA).

## 3. Results

### 3.1. Construction of a Functional TIA1-APEX2 Fusion Protein for Proximity Labeling

**Figure 1:**
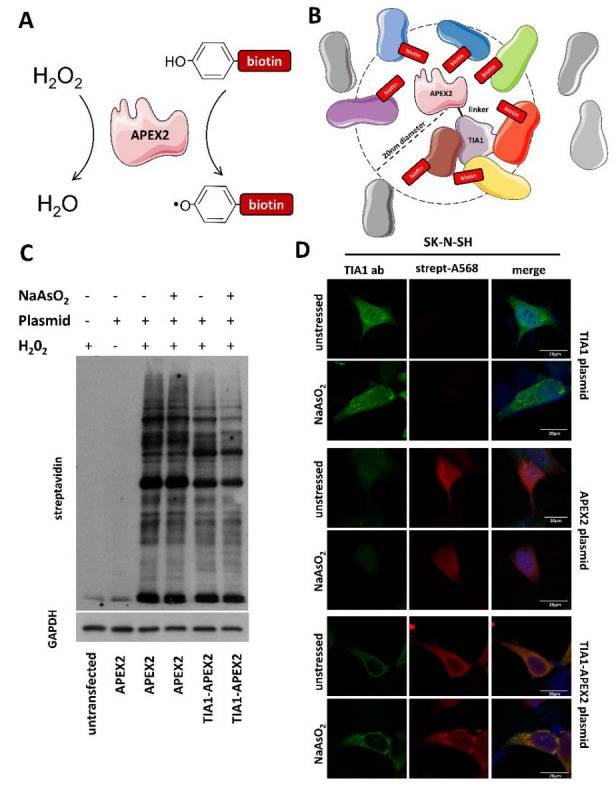
Proximity mapping of TIA1 interacting proteins by APEX2 mediated biotinylation. (**A**) H_2_O_2_ activates the APEX2 enzyme, which catalyzes biotin-phenol oxidation to generate a short-lived biotin phenoxyl radical that covalently tags endogenous proteins proximal to APEX2. (**B**) Schematic diagram of the TIA1-APEX2 proximity labeling depicting a genetically engineered APEX enzyme tagged via a linker sequence to the C-terminus of the wild-type human TIA1. Labeled proteins are within 20 nm from the APEX2; some will directly interact with TIA1. (**C**) Neutravidin-HRP Western blotting of induced protein biotinylation in lysates from APEX2 or TIA1-APEX2 expressing cells. (**D**) Immunostaining of unstressed and sodium arsenite-treated SK-N-SH cells transfected with either TIA1, APEX2, or TIA1-APEX2 plasmids. Subcellular localization is altered upon stress for TIA1 and TIA1-APEX2, but not APEX2. Please refer to the supplementary material for uncropped Western Blot images.

To construct the plasmid for proximity biotin labeling, the APEX2 DNA fragment was joined to TIA1 DNA at its C-terminus and inserted into a pAAV expression vector utilizing a CAGGS promoter and a WPRE sequence for efficient transcription and translation, respectively. A flexible (GGGS)3 linker sequence was also inserted between the two proteins to prevent the biological activity of the two subunits from being hampered. The fusion protein’s functionality was then examined. Neuroblastoma SK-N-SH cells were transfected with either the APEX2 alone control or the TIA1-APEX2 plasmids, and after 48 h, the cells were incubated with biotin phenol with or without sodium arsenite for 45 min before activating APEX2 with a short pulse of H_2_O_2_ to induce labeling. After lysing the cells, the biotinylated proteins were extracted with streptavidin magnetic beads (Figure 1A,B). Western blots probed with neutravidin-HRP confirmed APEX2 labeling using the APEX2 control plasmid, but not with the TIA1-APEX2 plasmid; when the nitrocellulose membrane was re-probed with TIA1 antibody, it was discovered that the fusion protein was cleaved (Supplementary Figure S1A,B). As a result, two new constructs were prepared, one with a different flexible linker (GSAGSAAGSGEF) and the other with a much longer rigid linker (GGAEAAAKEAAAKAAPAEAAAKEAAAKA). We repeated the experiment and discovered that these linker sequences preserved APEX2 activity while keeping the fusion protein status. However, while the plasmid with the flexible linker produced more protein staining, it was also substantially more cytotoxic; therefore, subsequent experiments were conducted using the TIA1-APEX2 plasmid with the rigid linker (Supplementary Figure S1A,C). As an additional step to identify if TIA1 subcellular mobilization and SG localization are preserved in the fusion protein, immunocytochemistry was carried out using an antibody against TIA1 and streptavidin-568 against biotin on SK-N-SH cells transfected with a TIA1 or the two APEX2 plasmids following acute stress. Figure 1D shows that while APEX2 labeling was diffuse in cells regardless of whether they were treated with sodium arsenite or not, TIA1 and TIA1-APEX2 distribution became progressively punctuated in response to stress, reminiscent of SG formation/localization.

### 3.2. Proteomic Identification of TIA1 Partners in Unstressed and Stressed Conditions

Proteomics analysis (LC-MS/MS) was next used to identify putative TIA1 interacting proteins in unstressed and acutely stressed (sodium arsenite, 45 min) neuroblastoma cells. Twelve biologically independent labeling experiments were carried out for each condition, then three replicates were combined, and four separate LC-MS/MS analyses were conducted. In total, 395 proteins were detected across TIA1-APEX2 experiments by LC-MS/MS in control conditions, and after filtering out the dataset for non-specific labeling by APEX2 (571 proteins), 86 proteins (20 in two or more replicates) were unique to TIA1 (Supplementary Table S2). In acutely stressed conditions, TIA1-APEX2 labeled 1.5 times as many proteins (562 proteins), and after filtering out the APEX2 dataset (446 proteins), 203 proteins (82 in two or more replicates) unique to TIA1 were identified (Supplementary Table S2).

### 3.3. Bioinformatic Analysis of TIA1 Partners in Unstressed and Stressed Conditions

**Figure 2:**
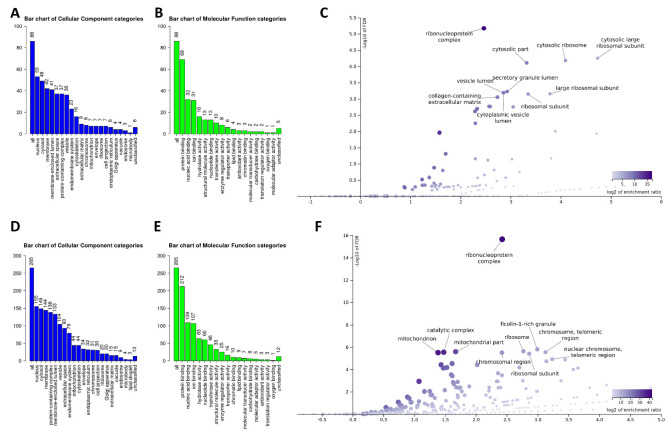
Gene Ontology ‘Cellular Component’ and ‘Molecular Function’ classification of TIA1 interacting proteins. ‘Cellular Component’ (blue bars) and ‘Molecular Function’ (green bars) categories of all TIA1 interacting partners in unstressed (**A**,**B**) and sodium arsenite-treated SK-N-SH cells (**D**,**E**). The height of the bar represents the number of protein IDs in the category. (**C**,**F**) ‘Cellular Component’ categories that are specifically enriched in unstressed and sodium arsenite-treated SK-N-SH cells, respectively. Both bar charts and Volcano plots were visualized using the WebGestalt analysis software 2019.

The WebGestalt gene set analysis program was used to evaluate the protein lists acquired from the proteomics studies. Figure 2A,B depict the Gene Ontology ‘Cellular Component’ and ‘Molecular Function’ of all TIA1 partners in unstressed settings. As shown, the identified proteins exhibited ‘protein binding’ and ‘nucleic acid binding’ capabilities and are evenly distributed between the ‘nucleus’ and the ‘cytoplasm’. Following enrichment analyses, TIA1-interacting partners were predominantly localized in ‘ribonucleoprotein complexes’ [Enrichment/Ratio (E/R) 5.5, FDR 5.7 × 10^−9^] and the ‘large ribosomal subunit’ (E/R 26, FDR 0.000056) (Figure 2C). The ‘Cellular Component’ and ‘Molecular Function’ of the TIA1 partners in stress conditions are depicted in Figure 2D,E, respectively. Proteins are distributed equally between ‘nucleus’ and ‘cytoplasm’, as they are in the unstressed condition, but two additional subcellular domains, ‘membrane’ and ‘protein-containing complex,’ are also prominently represented in stress conditions. Regarding ‘Molecular Function’, they too display ‘protein binding’ and ‘nucleic acid binding’ capabilities. Enrichment analysis of TIA1′s partners’ localization during stress revealed ‘ribonucleoprotein complexes’ (E/R 5.3, FDR 0], ‘ficolin-1-rich granule’ (E/R 8, FDR 0.0000015), and ‘ribosome’ (E/R 6.8, FDR 0.0000023) as the most significantly overrepresented categories (Figure 2F).

**Figure 3:**
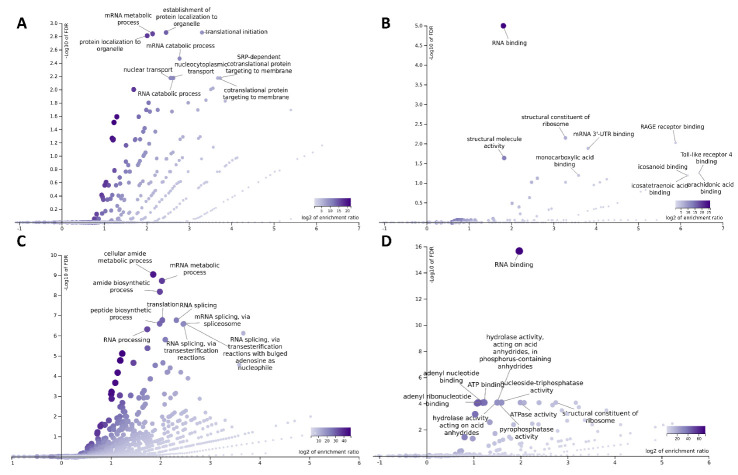
Gene Ontology Biological Process (left) and Molecular Function (right) categories enriched by TIA1 interacting proteins in unstressed (**A**,**B**) and sodium arsenite-treated (**C**,**D**) cells. Volcano plots were visualized by WebGestalt analysis software.

The Gene Ontology ‘Biological Processes’ and ‘Molecular Functions’ selectively enriched following TIA1-APEX2 labeling were also investigated by the WebGestalt toolkit. In unstressed conditions, TIA1-interacting partners were associated primarily with the ‘establishment of protein localization to organelle’ (E/R 5.5, FDR 0.0014), ‘translation initiation’ (E/R 9.9, FDR 0.0014), and ‘mRNA metabolic process’ (E/R 4.4, FDR0.0014). They mostly displayed ‘RNA binding’ (E/R 3.5, FDR 5.4 × 10^−9^) and proinflammatory ‘RAGE receptor binding’ activities (E/R 59, FDR 0.000015), or were ‘structural constituents of ribosomes’ (E/R 9.7, FDR 0.0000076) (Figure 3A,B). During acute stress, the TIA1-interacting partners were associated with the ‘cellular amide metabolic and biosynthetic process’ (E/R 3.6 FDR 9.3 × 10^−10^; E/R 4, FDR 6.7 × 10^−9^), ‘mRNA metabolic process’ (E/R 4.1, FDR 1.9 × 10^−9^), and ‘RNA splicing’ (E/R 5, FDR 1.7 × 10^−7^). Proteins displayed ‘RNA binding’ (E/R 3.9, FDR 0), ‘nucleoside-triphosphatase and ATPase activity’ (E/R 3, FDR 0.000084; E/R 4, FDR 0.000084) or were ‘structural constituents of ribosomes’ (E/R 6.7, FDR 0.000084) (Figure 3C,D). Based on these data, it is concluded that although only a few TIA1 partners were common in the two conditions (24 proteins, 8%), there is a high overlap in their overall function irrespective of the cellular state.

**Figure 4:**
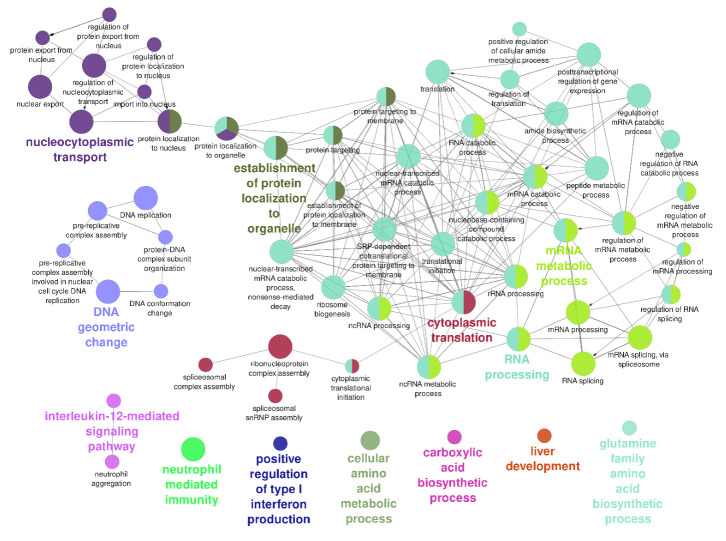
Interaction network of enriched biological processes obtained from the 265 unique proteins labeled in TIA1-APEX2 in both unstressed and sodium arsenite-treated cells. GO terms are represented as nodes, and the node size represents enrichment significance. The color of the nodes changes depending on the functional groupings and cluster distribution. The network was visualized in Cytoscape running the ClueGo plugin using a yFiles organic layout.

To visualize the enriched biological terms for both control and stressed conditions in a functionally grouped network, we pooled together the proteins from the two TIA1 interacting proteomes (86 + 203 = 289 proteins) and ran the ClueGo plugin for the 265 unique proteins on Cytoscape. Figure 4 shows the interconnection of the different processes as well as those that are not directly related to others. Most enriched categories involve RNA processes with 5 hubs: ‘RNA processing’, ‘RNA translation’, ‘RNA metabolism’, ‘protein localization’, and ‘nucleocytoplasmic transport’. Other categories such as immunological (‘Interleukin 12-mediated signaling’, ‘neutrophil-mediated immunity’, ‘positive regulation of type I interferon production’) and DNA (‘DNA geometry change’) processes stood alone and were not directly linked to RNA processes. Further, Table 1 categorizes TIA1-interacting proteins according to their enriched ‘Μolecular Function’. As shown, most proteins display ‘RNA binding’ and ‘DNA binding’ activities.

### 3.4. Proteomic Data Validation and Identification of TIA1 Directly Interacting Proteins

**Figure 5:**
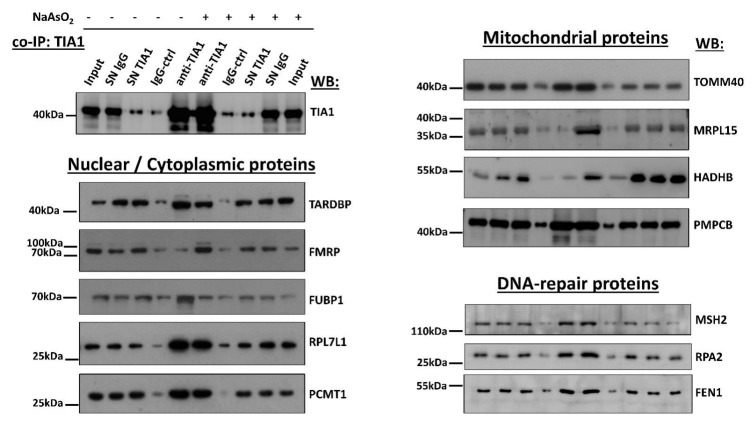
Several protein partners revealed by proximity biotinylation were proved to interact with TIA1 directly. TIA1-overexpressing SK-N-SH cells treated with or without sodium arsenite were subjected to co-immunoprecipitation reactions using anti-TIA1 antibody or IgG as a control. Precipitated proteins and the original lysates (inputs) were analyzed by SDS-PAGE followed by immunoblotting detecting TIA1, TARDBP (TDP43), FMRP FUBP1, RPL7L1, PCMT1, TOMM40, HADHB, PMPCB, MSH2, RPA2, and FEN1. SN: supernatant of the lysate after immunoprecipitation. Please refer to the supplementary material for uncropped Western Blot images.

To corroborate proteomics data, we conducted co-immunoprecipitation studies for selected proteins. We confirmed that TIA1 displayed a strong affinity for protein-L-isoaspartate O-methyltransferase (PCMT1), TARDBP, far upstream element binding protein 1 (FUBP1), fragile X mental retardation protein (FMRP), and ribosomal protein L7 Like 1 (RPL7L1). Further, TIA1 directly interacted with the mitochondrial proteins translocase of outer mitochondrial membrane 40 (TOMM40), mitochondrial ribosomal protein L15 (MRPL15), hydroxyacyl-CoA dehydrogenase trifunctional multienzyme complex subunit beta (HADHB), and peptidase mitochondrial processing subunit beta (PMPCB). TIA1 was also found to directly bind the DNA-repair proteins MutS homolog 2 (MSH2), Flap structure-specific endonuclease 1 (FEN1), and Replication protein A2 (RPA2) (Figure 5).

## 4. Discussion

TIA1 is a prototypical RBP that controls transcriptome and proteome diversity, as well as cellular stress response. Multiple elegant studies have revealed its regulatory roles in embryogenesis, differentiation, inflammation, viral infection, tumorigenesis, and neurodegeneration. These roles are mediated by its three N-terminal RNA recognition motifs (RRMs), and its C-terminal disordered prion-like domain (PrLDs) that supports multivalent protein-protein interactions. However, no study has captured TIA1′s interacting proteome until now. Here, the APEX2 labeling method was used to identify all of its interacting partners within a living cell environment (as opposed to a cell lysate), recognizing the importance of subcellular complexity and spatial context. TIA1 partners in control and acute stress conditions were characterized, and several partners were validated with co-immunoprecipitation. There are three points to be made here, all of which are related to the experimental approach and data interpretation. First, APEX2 labels any protein within approximately a 20 nm radius, not simply those that have been directly complexed with TIA1. This enabled the resolution of the molecular neighborhood, which is potentially more relevant than pinpointing direct partners. Second, a rigorous approach in capturing TIA1′s interacting proteome was used by subtracting all those proteins randomly pulled-down with APEX2 alone, rather than using an enrichment approach. Third, this study used a single ex vivo neuroblastoma population and a single stress stimulus for a specific length of time, which likely yielded only a subset of the potential proteome with which TIA1 interacts. In fact, a previous study has shown that as much as 60% of the SG proteome is different between cell types [30]. In addition, around 25% of SG-associated RBPs exhibit stress-type-specific SG targeting [30]. Another study corroborates these findings, demonstrating that different stresses recruit distinct RNAs onto SGs that have RNA binding sites enriched for only particular RBPs [31].

In terms of the actual data, there was a noticeable quantitative and qualitative difference between the proteomes obtained under control and stressed conditions. We found nearly three times as many proteins interacting with TIA1 during acute stress, which is perhaps expected given that TIA1 is an SG nucleation protein [11]. In the control condition, the TIA1 enriched network of proteins was almost exclusively associated with cytosolic processes related to mRNA metabolism, such as translation initiation, nucleocytoplasmic transport, catabolism, and protein localization. In the acute stress condition, TIA1 partners showed a broader subcellular distribution, including chromosomes and mitochondria. The latter finding, backed by co-immunoprecipitation of mitochondrial HADHB, PMPCB, TOMM40, and MRPL15 proteins, reiterates TIA1 role in mitochondrial dynamics and function (reviewed in [32]).

TIA1 protein partners during acute stress, similar to those in the control condition, regulate activities associated with mRNA metabolism, emphasizing splicing and translation. A potentially novel finding here is that TIA1 may influence protein synthesis not only by binding to U-rich motifs of mature mRNAs or other RBPs as has been widely reported, but also by interacting with translation initiation factors such as EIF2S2, EIF2S3, EIF3G, EIF3I, and a variety of ribosomal proteins. Moreover, during acute stress, TIA1 partners included DNA double-strand break (DSB) repair proteins such as CUL4B, FEN1, MSH2, RPA2, XRCC5, and XRCC6. Co-immunoprecipitation analysis revealed direct interaction of TIA1 with MSH2, FEN1, and RPA2. Interestingly, another study utilizing a multi-bait APEX2 approach for related RBPs (FMR1, FXR1, and G3BP1) also observed enrichment in proteins associated with DNA or chromatin [33]. These interactions are perhaps reminiscent of a property shown by the RBPs fused in sarcoma (FUS) and TDP43 in promoting DNA repair by concentrating DSB signaling and repair factors [34].

## 5. Conclusions

This study provided a system-level view of the TIA1 interactome and pinpointed its functional connectivity hubs in the cell. Mechanistic studies to determine the role of TIA1 in each of these processes may now be facilitated.

## Figures and Tables

**Table 1:** Enriched molecular functions of proteins labeled in TIA1-APEX2 reactions. The table provides the results of ClueGO molecular function analysis. Nr: number of genes associated with the GO term. %: genes identified as a proportion of total related genes in GO. PVal: *p*-value of the GO term after Benjamini-Hochberg correction.

GO Term	Nr	%	Associated Genes	PVal
RNA binding	89	5	*AGO2*, *AIMP1*, *ALDH18A1*, *ANKHD1*, *ANXA1*, *ARCN1*, *ASS1*, *CCAR1*, *CDC5L*, *CSTF2*, *CTNNA1*, *DCD*, *DDX20*, *DDX39B*, *DDX41*, *DHX30*, *DHX36*, *EIF2S2*, *EIF2S3*, *EIF3G*, *EIF3I*, *ESRP2*, *EXOSC10*, *FAU*, *FIP1L1*, *FMR1*, *FUBP1*, *HADHB*, *HNRNPA2B1*, *HNRNPH1*, *HNRNPH2*, *HNRNPL*, *HNRNPM*, *IGF2BP1*, *ILF2*, *ILF3*, *IMP4*, *IPO5*, *LARP1*, *LARP4*, *LGALS3*, *LRPPRC*, *LRRC47*, *MRPL15*, *MRPL4*, *MRPS34*, *MTREX*, *NAA15*, *NAP1L1*, *NME1*, *NPM1*, *PABPC3*, *PDCD4*, *PELP1*, *PPP1CC*, *PSMC1*, *PUF60*, *RPL10A*, *RPL12*, *RPL13A*, *RPL17*, *RPL27*, *RPL34*, *RPL37A*, *RPL39*, *RPL7A*, *RPL7L1*, *RPP25L*, *RPS15*, *RPS17*, *RTRAF*, *SART3*, *SF3B1*, *SLC4A1AP*, *SNRPD1*, *SRBD1*, *SRRM1*, *SRSF9*, *TARDBP*, *TIA1*, *TIAL1*, *TRMT1*, *TRMT10A*, *TXN*, *XRCC5*, *XRCC6*, *YWHAE*, *ZC3HAV1*, *ZNF622*	5.5 × 10^−26^
Translation initiation factor activity	5	10	*AGO2*, *EIF2S2*, *EIF2S3*, *EIF3G*, *EIF3I*	4.7 × 10^−2^
C-acyltransferase activity	4	14	*ACAT1*, *ACSM5*, *HADHB*, *SPTLC1*	3.8 × 10^−2^
Protein C-terminus binding	10	5	*AGO2*, *CCN2*, *CSK*, *MSH2*, *PHB*, *PPP1CC*, *PPP2CB*, *PRDX3*, *XRCC5*, *XRCC6*	3.9 × 10^−2^
Ubiquitin protein ligase binding	13	4	*CACYBP*, *CUL4B*, *EGFR*, *ERLIN2*, *LRPPRC*, *PSMD1*, *RELA*, *RPA2*, *RPL17*, *TPI1*, *TRAF2*, *XRCC5*, *YWHAE*	3.6 × 10^−2^
Cadherin binding	17	5	*ANXA1*, *CTNNA1*, *EGFR*, *EHD4*, *EIF2S3*, *EMD*, *GAPVD1*, *JUP*, *LARP1*, *PCMT1*, *PTPN11*, *PUF60*, *RPL34*, *RPL7A*, *S100A11*, *YWHAE*, *ZC3HAV1*	1.1 × 10^−3^
RAGE receptor binding	3	30	*S100A7*, *S100A8*, *S100A9*	2.1 × 10^−2^
pre-mRNA intronic binding	3	25	*HNRNPA2B1*, *HNRNPL*, *TARDBP*	3.6 × 10^−2^
Nucleocytoplasmic carrier activity	4	13	*CSE1L*, *IPO4*, *IPO5*, *KPNA5*	5.0 × 10^−2^
Ligase activity, forming carbon-carbon bonds	3	30	*KRT17*, *MCCC1*, *PCCA*	2.1 × 10^−2^
Organic acid binding	9	5	*ASS1*, *FABP5*, *GLUL*, *HBD*, *KRT17*, *MCCC1*, *PCCA*, *S100A8*, *S100A9*	3.8 × 10^−2^
Biotin binding	3	38	*KRT17*, *MCCC1*, *PCCA*	1.0 × 10^−2^
DNA helicase activity	9	10	*ANXA1*, *DHX36*, *MCM2*, *MCM4*, *MCM5*, *RAD50*, *WRNIP1*, *XRCC5*, *XRCC6*	3.0 × 10^−4^
Damaged DNA binding	6	8	*CUL4B*, *FEN1*, *MSH2*, *RPA2*, *XRCC5*, *XRCC6*	4.3 × 10^−2^
Telomeric DNA binding	5	11	*HNRNPA2B1*, *RAD50*, *RPA2*, *XRCC5*, *XRCC6*	3.1 × 10^−2^
Double-stranded telomeric DNA binding	3	30	*RAD50*, *XRCC5*, *XRCC6*	2.1 × 10^−2^
ATPase, acting on DNA	15	7	*ANXA1*, *DDX20*, *DDX39B*, *DDX41*, *DHX30*, *DHX36*, *MCM2*, *MCM4*, *MCM5*, *MSH2*, *MTREX*, *RAD50*, *WRNIP1*, *XRCC5*, *XRCC6*	2.0 × 10^−5^
Single-stranded DNA binding	12	9	*ANXA1*, *DHX36*, *FUBP1*, *HNRNPA2B1*, *LRPPRC*, *MCM2*, *MCM4*, *MCM5*, *MSH2*, *NME1*, *RAD50*, *RPA2*	2.2 × 10^−5^
Helicase activity	15	8	*ANXA1*, *DDX20*, *DDX39B*, *DDX41*, *DHX30*, *DHX36*, *MCM2*, *MCM4*, *MCM5*, *MSH2*, *MTREX*, *RAD50*, *WRNIP1*, *XRCC5*, *XRCC6*	3.8 × 10^−6^
Double-stranded RNA binding	6	9	*AGO2*, *DHX36*, *FMR1*, *IGF2BP1*, *ILF2*, *ILF3*	2.3 × 10^−2^
Single-stranded RNA binding	9	10	*AGO2*, *ANXA1*, *EXOSC10*, *FMR1*, *HNRNPH1*, *ILF3*, *LARP4*, *PABPC3*, *TIA1*	3.6 × 10^−4^
mRNA binding	22	7	*AGO2*, *CSTF2*, *DHX36*, *EIF2S2*, *ESRP2*, *FMR1*, *FUBP1*, *HNRNPA2B1*, *HNRNPL*, *HNRNPM*, *IGF2BP1*, *ILF3*, *LARP1*, *LARP4*, *PABPC3*, *RPL13A*, *SF3B1*, *SLC4A1AP*, *SRBD1*, *TARDBP*, *TIA1*, *TIAL1*	6.2 × 10^−8^
mRNA 3′-UTR binding	11	12	*DHX36*, *FMR1*, *HNRNPA2B1*, *IGF2BP1*, *ILF3*, *LARP1*, *LARP4*, *PABPC3*, *TARDBP*, *TIA1*, *TIAL1*	6.7 × 10^−6^
mRNA 5′-UTR binding	4	14	*DHX36*, *FMR1*, *IGF2BP1*, *LARP1*	4.1 × 10^−2^
Poly-purine tract binding	4	14	*FMR1*, *LARP4*, *PABPC3*, *TIA1*	4.1 × 10^−2^
mRNA 3′-UTR AU-rich region binding	4	16	*DHX36*, *ILF3*, *TIA1*, *TIAL1*	2.6 × 10^−2^

The TIA1 interaction with six of the above proteins was also validated using co-immunoprecipitation in HEK293 cells (Figure S2). Nine other proteins, PHB2, IGF2BP1, MCCC1, SMC3, HSD17B4, PRDX3, PCCA, MCM2/4, and CUL4B, were not co-immunoprecipitated with TIA1 in SK-N-SH cells, indicating that their interaction is indirect/proximal or weak (not shown).

## Data Availability

Data is contained within the article or Supplementary Materials.

## Supplementary Materials

The following are available online at https://www.mdpi.com/article/10.3390/biology11020287/s1, Table S1. Primer sequences. Figure S1. Proximity mapping of TIA1 interacting proteins using TIA1-APEX2 constructs with different linker sequences. (A) SDS-PAGE and Neutravidin blotting of biotinylated lysates from APEX2 or TIA1-APEX2 expressing cells. Of note, the TIA-APEX2-flexible1 construct did not display APEX2 activity. (B) Western blotting for TIA1 in the same lysates. The cells transfected with the TIA1-APEX2-flexible1 plasmid expressed a protein in the endogenous TIA1 range (~42 kDa) while TIA-APEX2-rigid and TIA1-APEX2-flexible2 plasmids expressed a chimeric protein in the expected ~75 kDa range (APEX2 has a molecular weight of 27 kDa). Table S2. Complete lists of pulled-down proteins identified by LC-MS/MS analysis (FDR 0.01). Figure S2. Protein partners identified to directly interact with TIA1 in SK-N-SH, were also confirmed using Co-IP to interact directly with TIA1 in HEK293 cells.
