# Supplementary material for: In Situ Peroxidase Labeling Followed by Mass-Spectrometry Reveals TIA1 Interactome
Source: Biology (Basel). 2022 Feb 11;11(2):287. doi: 10.3390/biology11020287 (PMC8869308; doi:10.3390/biology11020287)
Supplement: Supplementary file 1 [file biology-11-00287-s001.zip › Full Western Blot/Figure 5 raw.pdf]

RPL7L1: lab book #4 p. 32

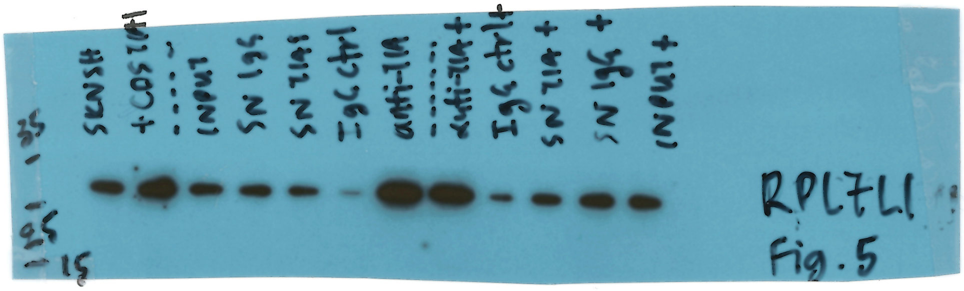

TOMM40: lab book #4 p. 33

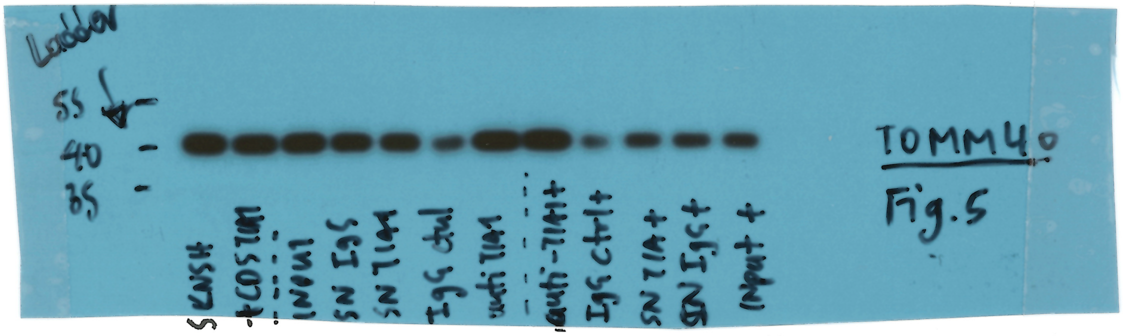

PCMT1 : lab book #4 p. 10

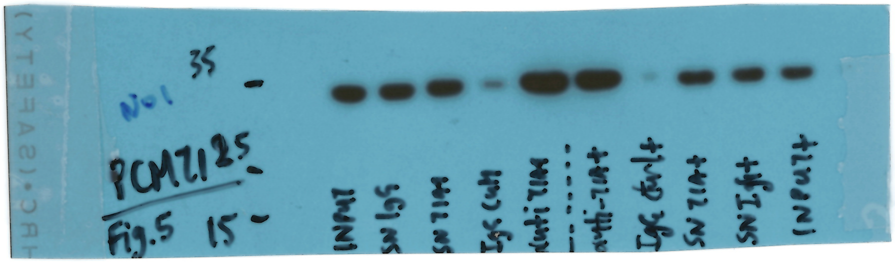

TDP43: lab book #3 p. 120

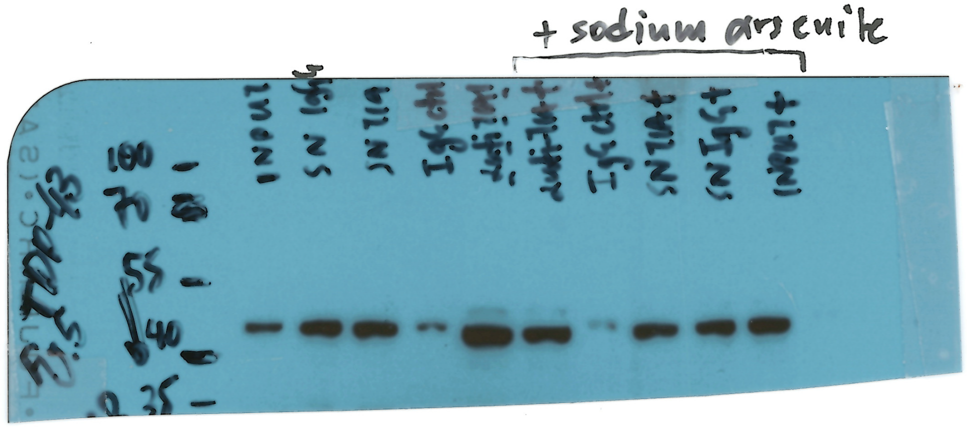

FMRP: lab book #4 p. 40

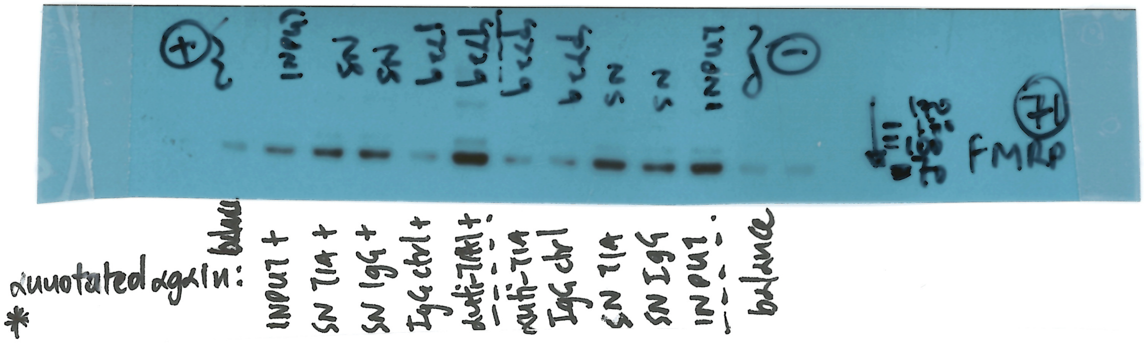

FUBP1 : lab book #4 p. 34

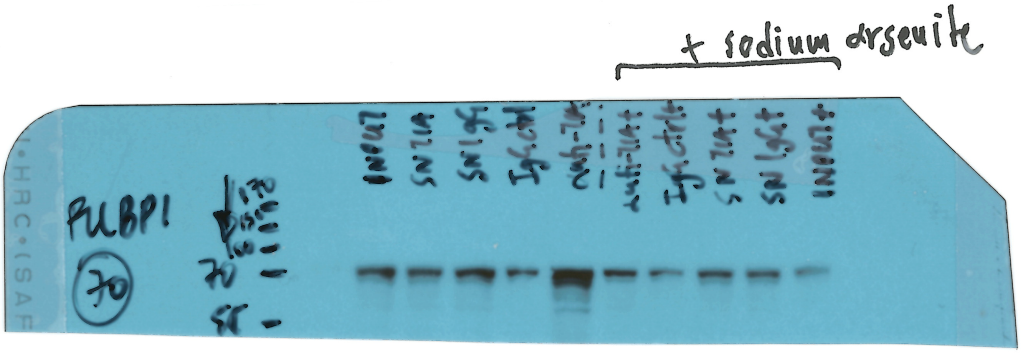

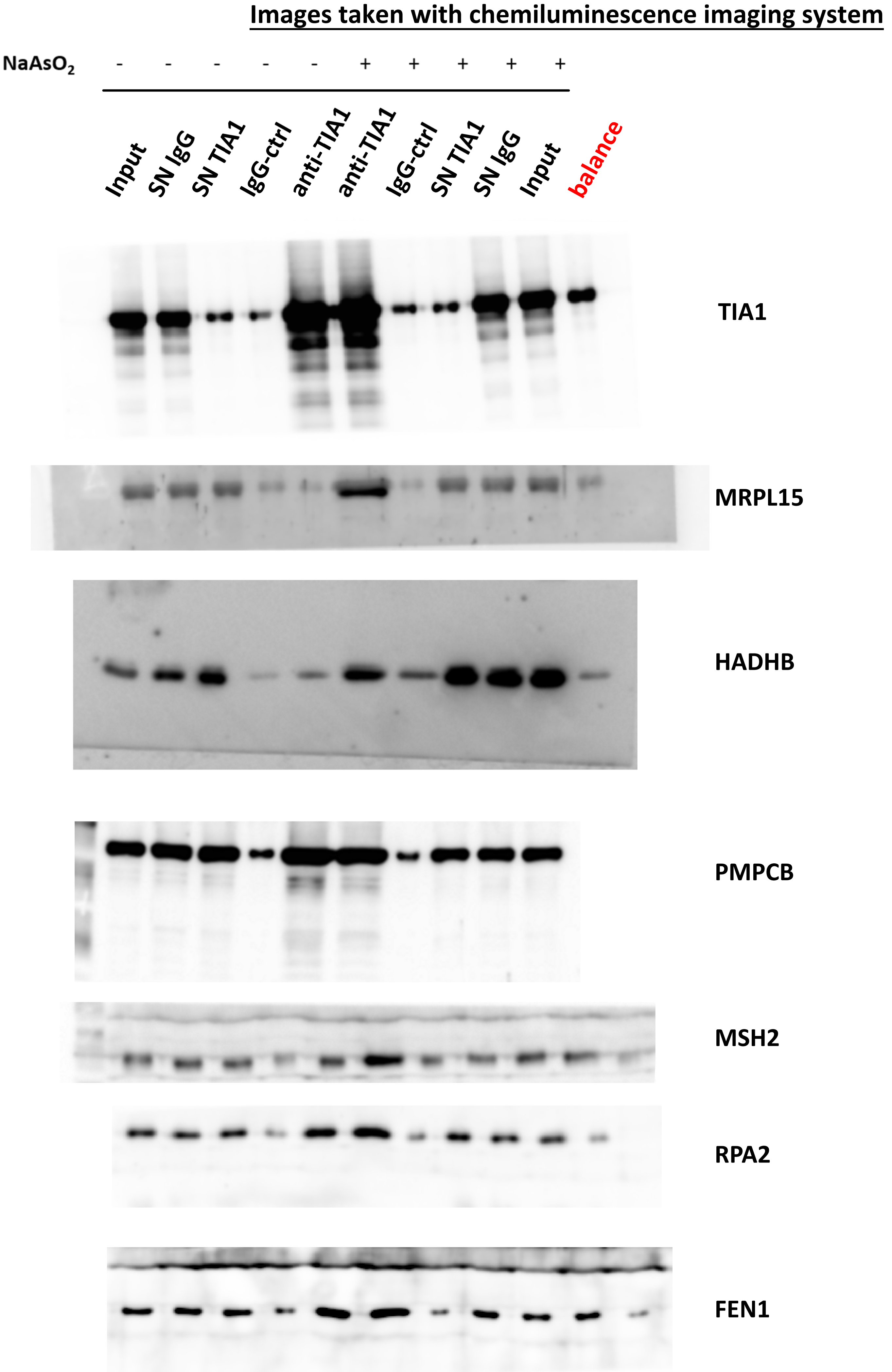
Images taken with chemiluminescence imaging system
$NaAsO_2$
- - - - - + + + + +
Input
SN IgG
SN TIA1
IgG-ctrl
anti-TIA1
anti-TIA1
IgG-ctrl
SN TIA1
SN IgG
Input
balance
TIA1
MRPL15
HADHB
PMPCB
MSH2
RPA2
FEN1
