# Supplementary material for: In Situ Peroxidase Labeling Followed by Mass-Spectrometry Reveals TIA1 Interactome
Source: Biology (Basel). 2022 Feb 11;11(2):287. doi: 10.3390/biology11020287 (PMC8869308; doi:10.3390/biology11020287)

Image taken with chemiluminescence imaging system

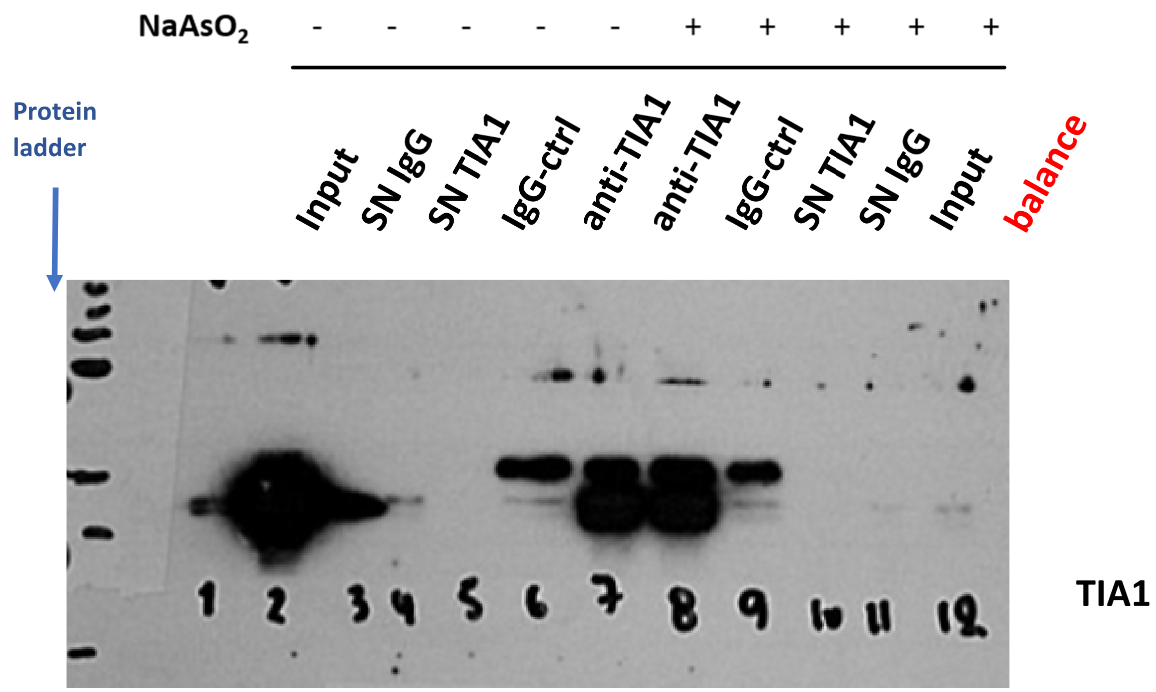

Lane annotated as no1: balance

Lane annotated as no2: overexpression of TIA1 in HEK293 cells from a different experiment to check if the transfection in co-immunoprecipitation experiment was successful.

Lane annotated as no3: although more concentration of sample 'input' seems to be loaded, in the reality is being affected from the sample in lane 2 (over-saturated)

Images taken with chemiluminescence imaging system

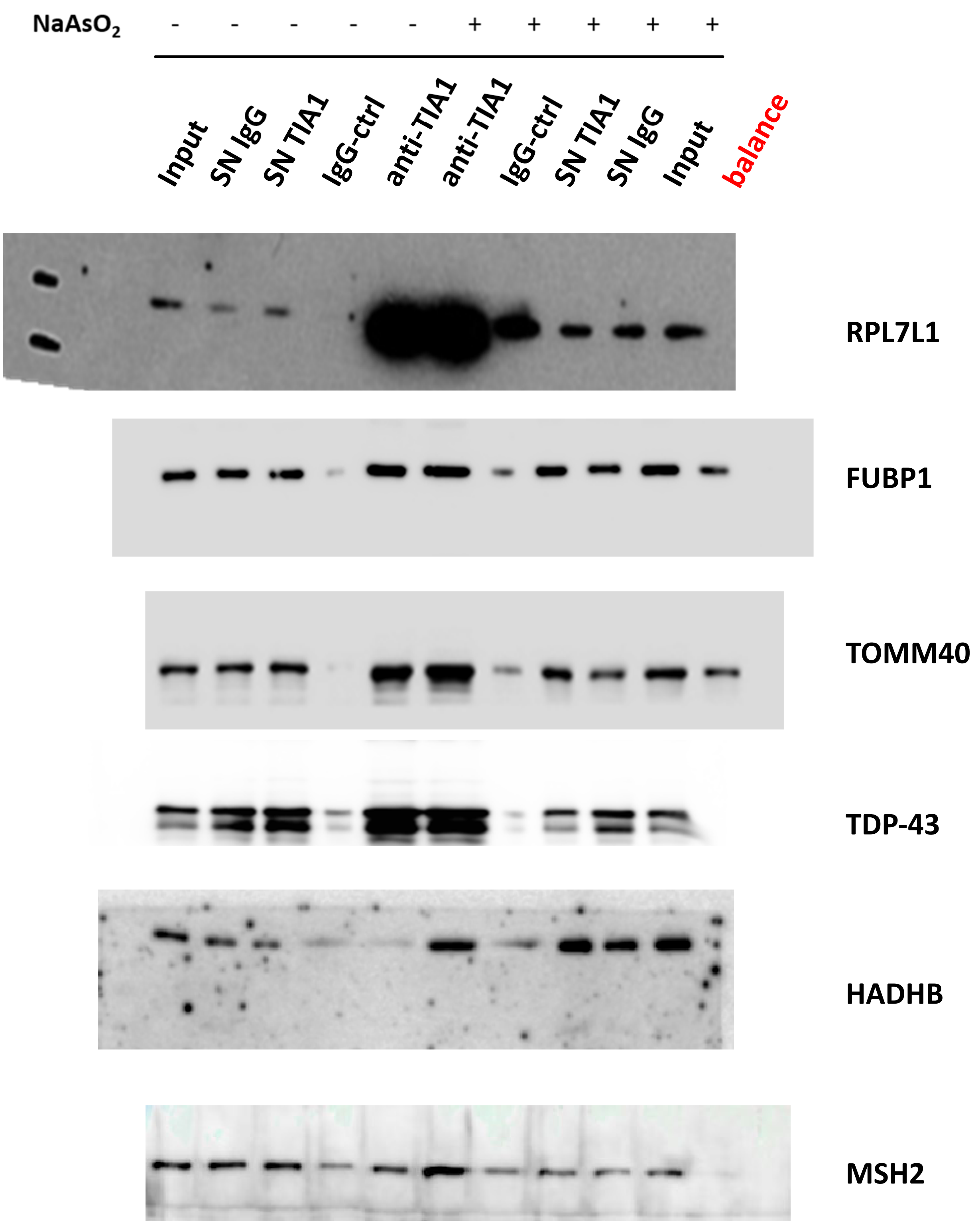

Supplement: Supplementary file 1 [file biology-11-00287-s001.zip › Full Western Blot/Figure S2 raw.pdf]
