# Supplementary material for: In Situ Peroxidase Labeling Followed by Mass-Spectrometry Reveals TIA1 Interactome
Source: Biology (Basel). 2022 Feb 11;11(2):287. doi: 10.3390/biology11020287 (PMC8869308; doi:10.3390/biology11020287)
Supplement: Supplementary file 1 [file biology-11-00287-s001.zip › Table S1.pdf]

Table S1. Primer sequences.

Primer set	Forward	Reverse
Flag_APEX2_NES_for_AAV	ATCATTTTGGCAAAGAATTGGATCCACCGCCACCATGGACTAC	CCAGAGGTTGATTATCGATAAGCTTCTATTAGTCCAGGGTCAGGC
Hs_TIA1_CDS_for_APEX2_in_AAV_Flexible 2	ATCATTTTGGCAAAGAATTGGATCCACCGCCACCATGGAGGACGAGATGCCC	GGAGCCAGCGGATCCCTGGGTTTCATACCCTGC
APEX2_CDS_for_TIA1_in_AAV_Flexible 2	TGAAACCCAGGGATCCGCTGGCTCCGCTGCTGGTTCTGGCGAATTCGGAAAGTCTTACCCAACTGTG	CCAGAGGTTGATTATCGATAAGCTTCTATTAGGCATCAGCAAACCCAAG
Hs_TIA1_CDS_for_APEX2_in_AAV_Rigid	ATCATTTTGGCAAAGAATTGGATCCACCGCCACCATGGAGGACGAGATGCCC	TGCTTCTGCGCCACCCTGGGTTTCATACCCTGC
APEX2_CDS_for_TIA1_in_AAV_Rigid	TGAAACCCAGGGTGGCGCAGAAGCAGCAGCAAAAGAAGCAGCAGCAAAAGCAGCACCGGGAAAGTCTTACCCAACTGTG	CCAGAGGTTGATTATCGATAAGCTTCTATTAGGCATCAGCAAACCCAAG
hs_TIA1_CDS_for_APEX2_in_AAV_Flexible 1	ATCATTTTGGCAAAGAATTGGATCCACCGCCACCATGGAGGACGAGATGCCC	TGAACCGCCTCCACCCTGGGTTTCATACCCTGC
APEX2_CDS_for_TIA1_in_AAV_Flexible 1	TGAAACCCAGGGTGGAGGCGGTTCAGGCGGAGGTGGCTCTGGCGGTGGCGGATCGGGAAAGTCTTACCCAACTGTG	CCAGAGGTTGATTATCGATAAGCTTCTATTAGGCATCAGCAAACCCAAG
hs_TIA1_CDS	ATCATTTTGGCAAAGAATTGGATCCACCGCCACCATGGAGGACGAGATGCCC	CCAGAGGTTGATTATCGATAAGCTTAGTCCTTATTCACTGGGTTTCATAC
