# Supplementary figures and images for: In Situ Peroxidase Labeling Followed by Mass-Spectrometry Reveals TIA1 Interactome
Source: Biology (Basel). 2022 Feb 11;11(2):287. doi: 10.3390/biology11020287 (PMC8869308; doi:10.3390/biology11020287)

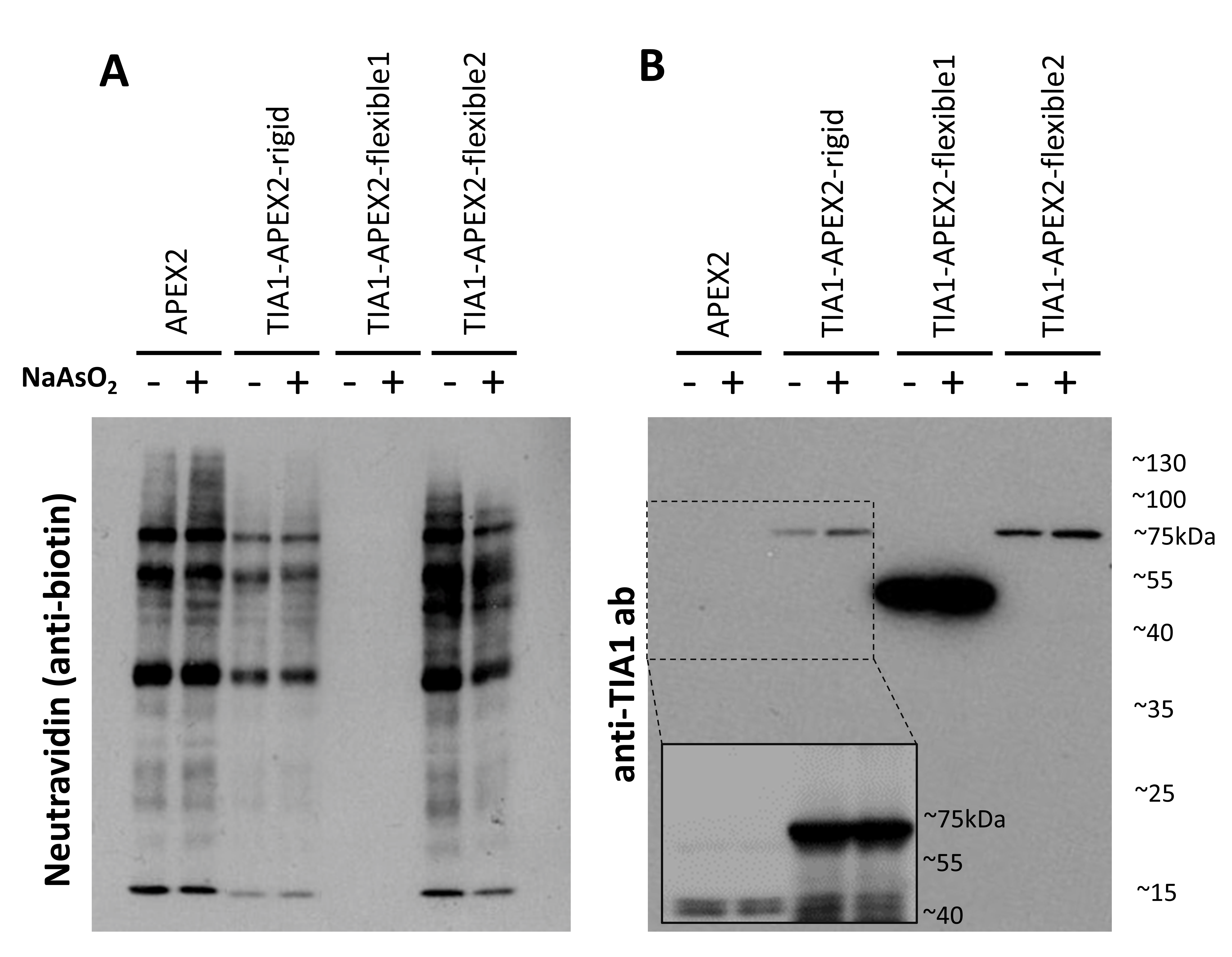

Supplement: Supplementary file 1 [file biology-11-00287-s001.zip › Figure S1.tif]

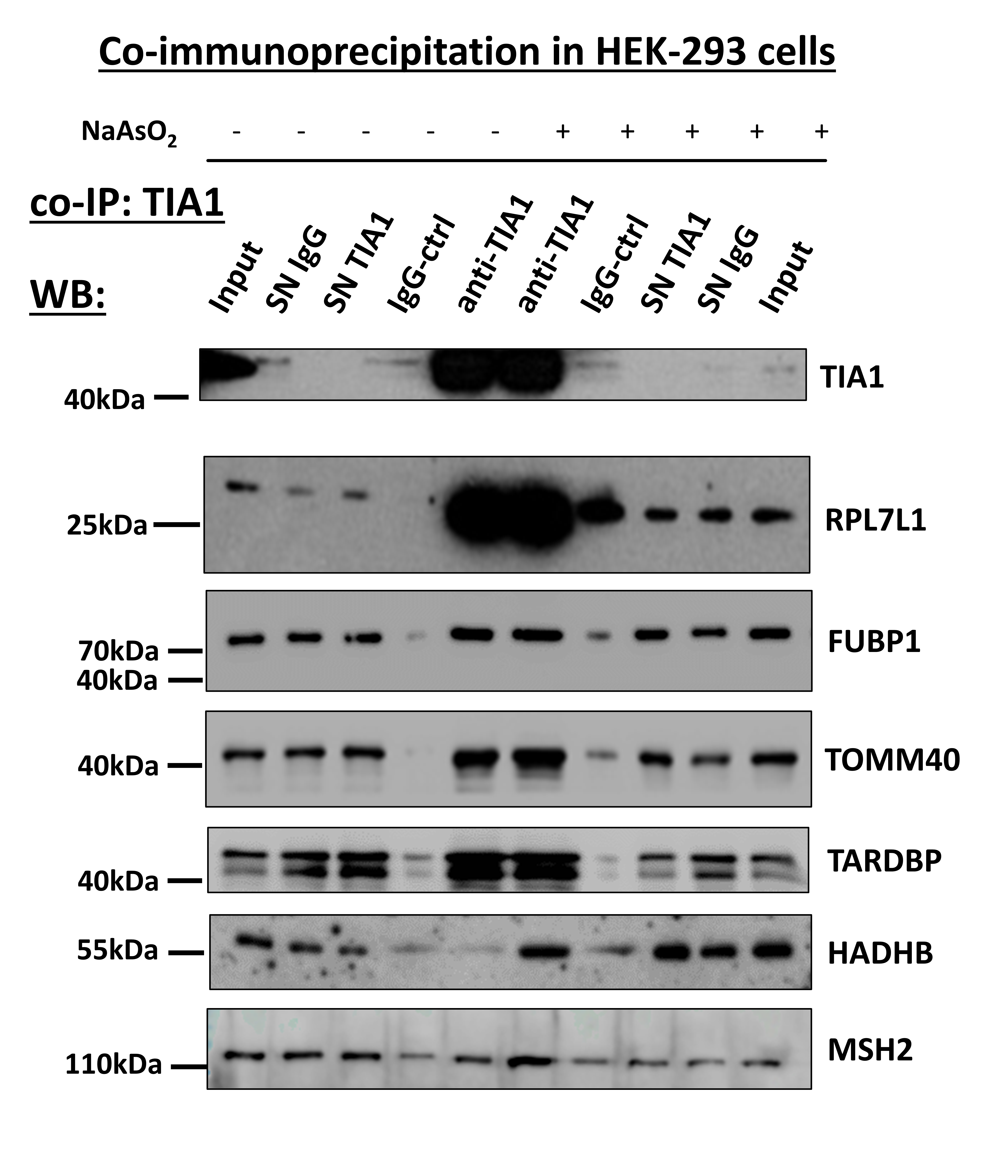

Supplement: Supplementary file 1 [file biology-11-00287-s001.zip › Figure S2.tif]

Fig. 1

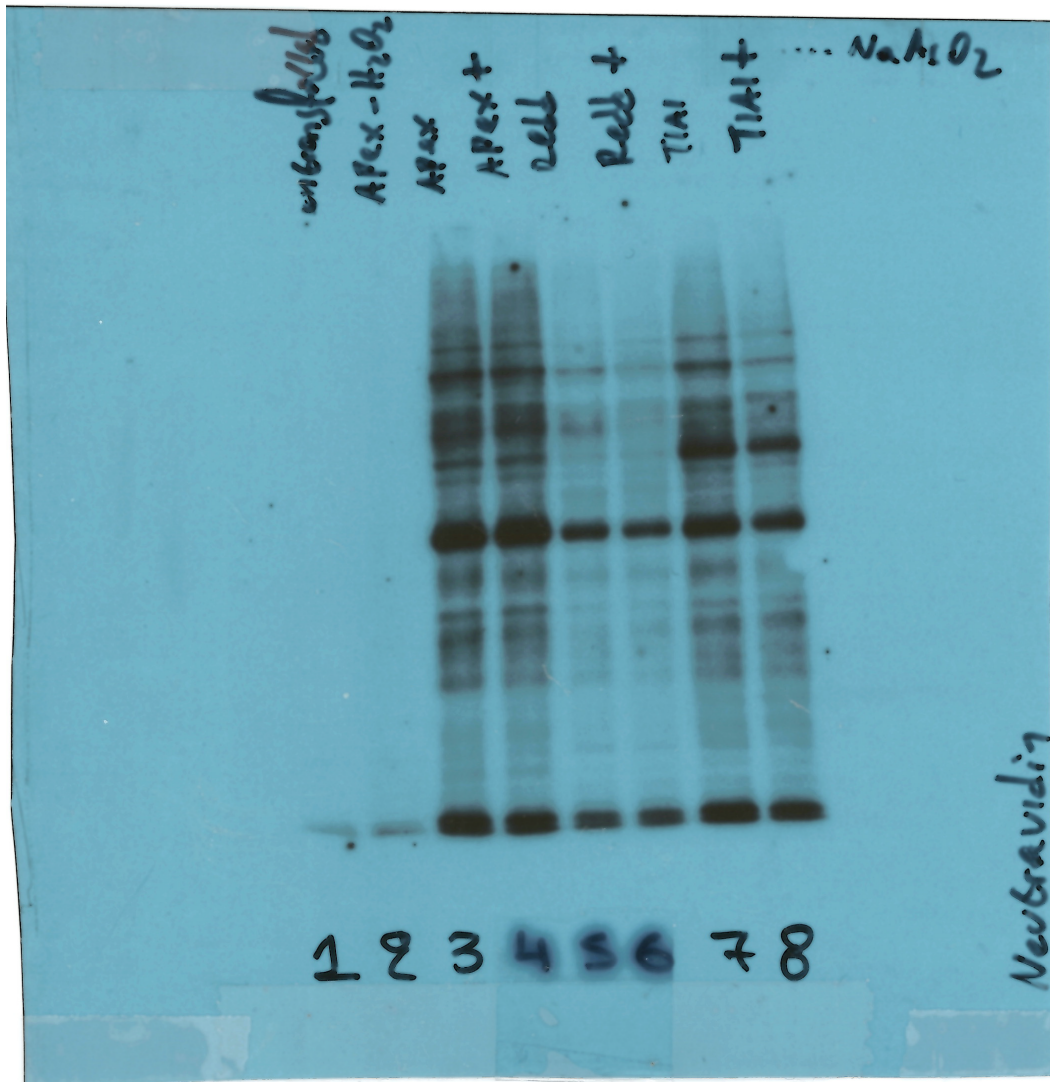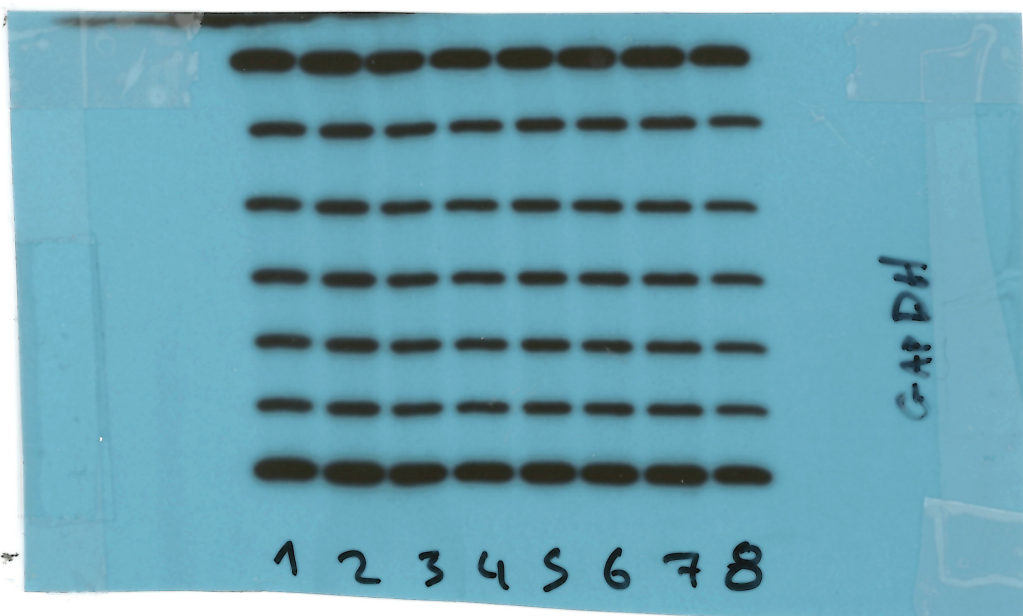

Supplement: Supplementary file 1 [file biology-11-00287-s001.zip › Full Western Blot/Figure 1 raw.pdf]

# Supplementary fig.1

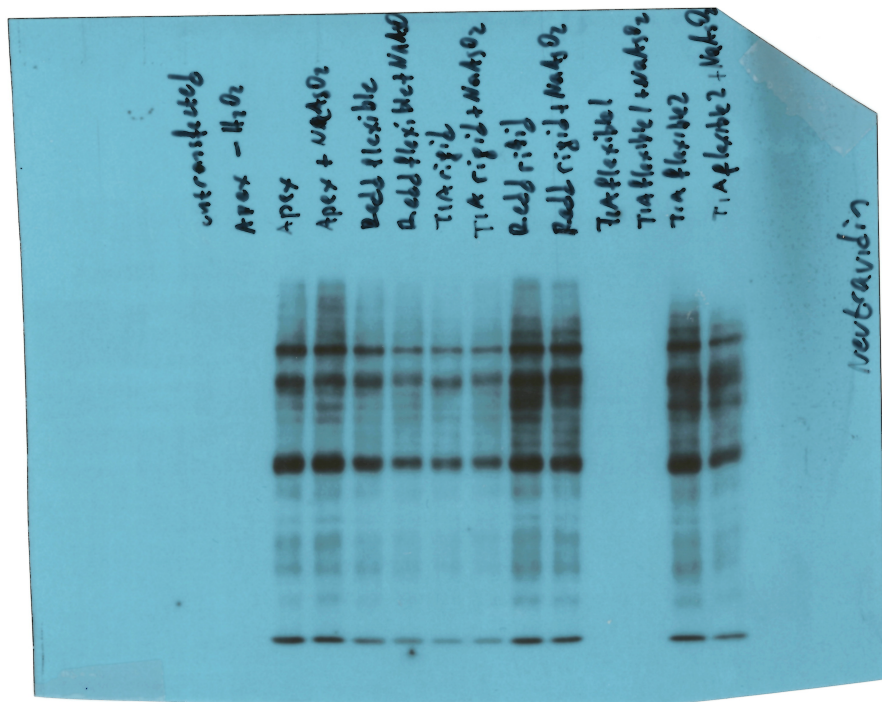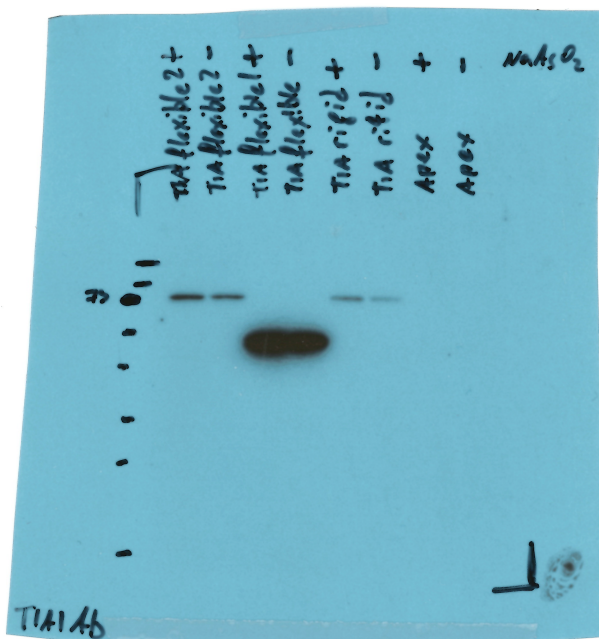

Image taken with chemiluminescence imaging system

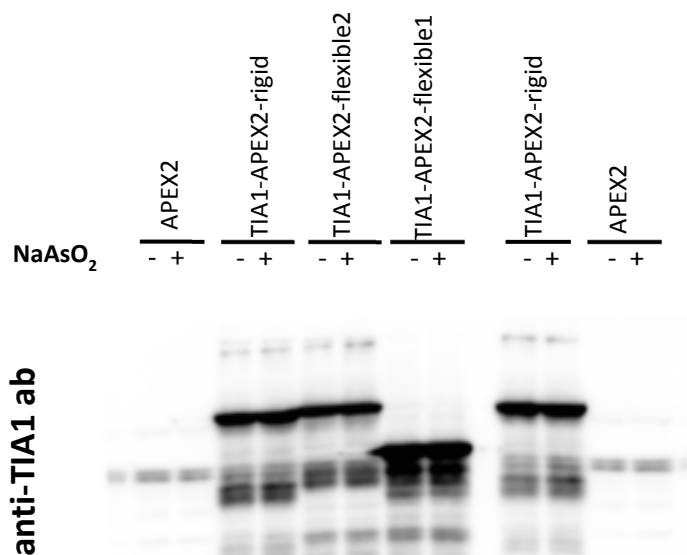

Supplement: Supplementary file 1 [file biology-11-00287-s001.zip › Full Western Blot/Figure S1 raw.pdf]
